# The role of supplementary environmental surveillance to complement acute flaccid paralysis surveillance for wild poliovirus in Pakistan – 2011–2013

**DOI:** 10.1371/journal.pone.0180608

**Published:** 2017-07-25

**Authors:** Tori L. Cowger, Cara C. Burns, Salmaan Sharif, Howard E. Gary, Jane Iber, Elizabeth Henderson, Farzana Malik, Syed Sohail Zahoor Zaidi, Shahzad Shaukat, Lubna Rehman, Mark A. Pallansch, Walter A. Orenstein

**Affiliations:** 1 Rollins School of Public Health, Emory University, Atlanta, Georgia, United States of America; 2 Division of Viral Diseases, National Center for Immunization and Respiratory Diseases, Centers for Disease Control and Prevention (CDC), Atlanta, Georgia, United States of America; 3 WHO Regional Reference Laboratory for Polio Eradication Initiative, Department of Virology, National Institute of Health (NIH), Islamabad, Pakistan; 4 Global Immunization Division, Center for Global Health, Centers for Disease Control and Prevention (CDC), Atlanta, Georgia, United States of America; 5 Department of Virology, National Institute of Health (NIH), Islamabad, Pakistan; 6 Division of Infectious Diseases, Emory University School of Medicine, Atlanta, Georgia, United States of America; The Scripps Research Institute, UNITED STATES

## Abstract

**Background:**

More than 99% of poliovirus infections are non-paralytic and therefore, not detected by acute flaccid paralysis (AFP) surveillance. Environmental surveillance (ES) can detect circulating polioviruses from sewage without relying on clinical presentation. With extensive ES and continued circulation of polioviruses, Pakistan presents a unique opportunity to quantify the impact of ES as a supplement to AFP surveillance on overall completeness and timeliness of poliovirus detection.

**Methods:**

Genetic, geographic and temporal data were obtained for all wild poliovirus (WPV) isolates detected in Pakistan from January 2011 through December 2013. We used viral genetics to assess gaps in AFP surveillance and ES as measured by detection of ‘orphan viruses’ (≥1.5% different in VP1 capsid nucleotide sequence). We compared preceding detection of closely related circulating isolates (≥99% identity) detected by AFP surveillance or ES to determine which surveillance system first detected circulation before the presentation of each polio case.

**Findings:**

A total of 1,127 WPV isolates were detected by AFP surveillance and ES in Pakistan from 2011–2013. AFP surveillance and ES combined exhibited fewer gaps (i.e., % orphan viruses) in detection than AFP surveillance alone (3.3% vs. 7.7%, respectively). ES detected circulation before AFP surveillance in nearly 60% of polio cases (200 of 346). For polio cases reported from provinces conducting ES, ES detected circulation nearly four months sooner on average (117.6 days) than did AFP surveillance.

**Interpretation:**

Our findings suggest ES in Pakistan is providing earlier, more sensitive detection of wild polioviruses than AFP surveillance alone. Overall, targeted ES through strategic selection of sites has important implications in the eradication endgame strategy.

## Introduction

In 1988, the World Health Assembly resolved to eradicate poliovirus. Since then, polio incidence has decreased from an estimated 350,000 cases to 37 in 2016 [1, 2]. A cornerstone of global eradication efforts and the primary means for detecting poliovirus transmission is acute flaccid paralysis (AFP) surveillance–clinical surveillance for persons with AFP, and subsequent testing for polioviruses in stool specimens [3, 4].

More than 99% of poliovirus infections occur without paralysis and therefore are not detected by AFP surveillance [5–8]. A person infected with poliovirus can excrete millions of infectious viruses in stool daily for several weeks to months after infection [9]. Since polioviruses are relatively stable in aqueous environments, testing for polioviruses in sewage, or environmental surveillance (ES), has the potential to detect polioviruses circulating in the community without relying on clinical presentation of disease as does AFP surveillance.

Previous evidence from simulation models suggests that when the paralysis-to-infection ratio is low (<1:200), ES may be more efficient than AFP surveillance in detecting circulating poliovirus, especially in areas with high vaccination coverage with inactivated polio vaccine (IPV) [10]. Evidence to support ES as a supplement to AFP surveillance has been described in case reports from Finland, Israel and the Palestinian Authority, Egypt, and India, and ES has contributed to documenting the elimination of wild poliovirus (WPV) from Egypt and India [11–22]. However in these settings, ES was implemented during time periods with limited poliovirus circulation with few or no reported polio AFP cases, making it difficult to quantitatively ascertain the impact of ES as a supplement to AFP surveillance. In contrast, Pakistan has implemented an extensive ES network during a period of continued endemicity, resulting in robust genetic sequence data for isolates detected by both ES and AFP surveillance over the same time period.

ES in Pakistan has consistently yielded positive isolates and informed actions to enhance polio control measures [11, 23]. In this analysis, we aimed to quantitatively investigate the role of ES as a supplement to AFP surveillance using national surveillance data from Pakistan, which includes genetic sequence data for all reported polio AFP cases and all ES isolates collected during 2011–2013. Specifically, we aimed to use viral genetics and molecular epidemiology to determine if supplemental ES 1) improves completeness and quality of surveillance, and 2) provides early warning of circulating poliovirus compared to AFP surveillance alone. With the addition of supplemental ES, if ES is beneficial we would expect to observe 1) fewer gaps in the viral phylogeny as measured by detection of fewer ‘orphan’ isolates (defined below) and 2) prior to the onset of each polio case, genetically-similar isolates would be detected by ES sooner on average than genetically-related isolates detected by AFP surveillance.

## Methods

### Study setting

In Pakistan, ES started in 2009 in two cities in two provinces–Karachi, Sindh Province and Lahore, Punjab Province. ES was then expanded during 2010 to 2012, with 23 sampling sites in 4 of the 6 provinces by the end of 2012 (S1 Fig) [11]. During the period of this study, 6 sites were added in 2012. ES sites were selected with input from NIH Pakistan and maintained as part of routine surveillance for poliovirus, and therefore, specific permissions for ES sites were not required. ES site locations were selected based on perceived risk for polio circulation, which was based on a combination of factors: 1) sustained transmission of polio previously noted in the district; 2) available and appropriate sewage systems; 3) general poor sanitation and crowding; 4) insufficient vaccination coverage; and 5) suspicion of sub-optimal AFP surveillance [11].

### Sample collection, processing, and data curation

Environmental sampling was conducted via monthly grab sampling from sewage systems in accordance with WHO’s Guidelines for Environmental Surveillance for Polioviruses [24]. All stool samples from AFP surveillance and ES sewage samples were transferred to the WHO Regional Reference Laboratory for Polio Eradication Initiative, Department of Virology, National Institute of Health, Islamabad, Pakistan and processed for virus isolation using the recommended methods of the WHO’s Global Poliovirus Laboratory Network [25, 26]. Wild poliovirus isolates were tested using reverse transcription-PCR to determine the serotype and to differentiate between wild and vaccine-derived polioviruses [27]. Isolates were then subjected to partial genomic sequencing of the complete VP1 capsid gene (906 nucleotides) and phylogenetic analysis as previously described [17, 26–28]. Isolates included in this analysis are routinely collected and used for surveillance and reporting of poliovirus in Pakistan, and the analysis herein aims to reframe these data to answer our specific research questions described above. For this analysis, we considered all WPV isolates collected nationwide through AFP surveillance and ES in Pakistan from January 2011 through December 2013. AFP isolates included both those from polio cases and their contacts. For ES isolates, genetically identical (i.e., duplicate) isolates from the same environmental sampling batch were removed, however genetically unique isolates from the same batch were included (i.e., multiple isolates per sampling batch included if their VP1 sequences were different).

### Orphan analysis

We first aimed to compare the overall completeness and quality of supplemental ES to AFP surveillance alone by looking for gaps in viral phylogeny manifested by the detection of ‘orphan viruses.’ Orphan viruses are defined as isolates that are ≥1.5% different in the VP1 capsid nucleotide sequence from any isolate previously detected, and their frequency is used as a surrogate measure of quality of surveillance (i.e., with good surveillance, each isolate is expected to be closely related to its progenitors). The poliovirus genome region analyzed diverges at approximately 1% nucleotide substitutions per year, and isolation of an orphan virus suggests silent circulation without detection for an extended period of time, indicating potential gaps in surveillance [29–31].

We used a MatLab software (The MathWorks, Natick, MA) script on all Pakistan WPV isolates to chronologically identify orphan viruses separately for three surveillance scenarios: 1) AFP surveillance alone; 2) ES alone; and 3) AFP surveillance plus ES. We compared the number of orphan isolates detected as a proportion of total isolates for each surveillance scenario to determine overall completeness and to determine what percentage of AFP orphans could be linked through ES and vice versa (e.g., if an orphan virus is detected when considering only AFP isolates, but not considered an orphan when considering both AFP and ES isolates, that virus is considered to be phylogenetically linked by the ES isolate).

### Circulation analysis

Secondly, we aimed to assess whether supplemental ES provides early warning of circulating poliovirus compared to AFP surveillance alone. To determine the proportion of poliovirus transmission that would have been detected sooner by ES than with AFP surveillance alone, we aimed to quantify detection time of genetically-similar circulating virus before and after each polio case to determine which surveillance system, ES or AFP surveillance, detected circulation earlier.

In total, 361 WPV1 AFP cases were reported in Pakistan during 2011–2013. Of these, 333 case isolates were genetically unique when compared to any other AFP isolate (defined as having at least one nucleotide difference from any other AFP isolate in VP1 capsid sequence), 22 polio case isolates had identical sequences (100% identity in VP1 capsid nucleotide sequence) to one other isolate (11 pairs), and six had identical sequences to two other isolates (2 triplets). We considered only the case isolate with the earliest symptom onset date from each pair or triplet and excluded subsequent identical isolates to eliminate duplication, resulting in 346 genetically unique polio case isolate sequences for analysis.

For each of these genetically unique polio case isolates, we used a MatLab script to generate a list of ‘genetically-similar’ viruses among both ES and AFP surveillance isolates, circulating before the onset of paralysis of each. We defined ‘genetically-similar’ surveillance isolates as isolates with ≥ 99.0% identity in VP1 capsid nucleotide sequence to the polio case isolate; this created 346 sets of isolates for analysis. Genetically-similar (≥99.0% identity) circulating ES and AFP surveillance isolates detected for one polio case isolate could also be matches for other polio cases if the surveillance isolate was genetically intermediate between the two polio cases.

For each polio case, we plotted the case isolate and their genetically-similar (≥99.0% identity) circulating surveillance isolates temporally, as depicted by the example in S2 Fig. We then calculated the 1) proportion of polio cases with preceding circulation detected by AFP surveillance only, ES only, both AFP surveillance and ES, or neither surveillance system; 2) type of surveillance system first detecting circulation for each polio case; 3) the length of time during which poliovirus circulation was detected by each respective surveillance system prior to each polio case (i.e., time from detection of the first genetically-similar surveillance isolate to symptom onset date of the polio case). We chose to analyze circulation before polio AFP isolates rather than before and after ES isolates for this analysis, since we were interested in the circulation detected before presentation of paralytic cases, and AFP surveillance represents the universally accepted reference standard. The unit of analysis for subsequent analyses was genetically unique polio case isolates (N = 346), and all statistical analyses were completed in SAS® (Version 9.3, Cary, NC). Polio cases could not be considered statistically independent because genetically-similar (≥99.0% identity) circulating ES and AFP surveillance isolates detected for one polio case could also be detected for other polio cases. Therefore, statistical tests were not conducted.

In Pakistan, two of six provinces were not conducting ES during this period and relied solely on AFP surveillance; we assumed polio cases reported from these provinces were less likely to have genetically-similar isolates detected by ES than cases that occur in areas nearer to environmental sampling sites. To account for this, polio cases were stratified by whether they occurred in a province conducting ES (i.e., Balochistan, Sindh, Punjab and Khyber Pakhtunkhwa) or reported from a province not currently conducting ES (i.e., Gilgit-Baltistan, and FATA). For polio cases reported from provinces not conducting ES, detection of genetically-similar circulating isolates by ES was possible if detection occurred at an ES site in a different province from where the polio case was reported; for example, a polio case reported from FATA may have a genetically-similar ES isolate detected at sampling site in Karachi. Finally, we calculated the measures described above to compare performance of ES between provinces conducting ES as compared to provinces not conducting ES. Complete depiction of circulation detected by ES and AFP surveillance for all 346 polio cases is shown in S3 Fig and a phylogenetic tree for a subset of isolates can be found in Alam et Al, Figure 3, 2014 [28].

## Results

### Descriptive analysis

A total of 366 AFP WPV isolates including 36 isolates from contacts of AFP cases and 761 ES WPV isolates from 291 environmental batches (Median: 2, range: 1–6 unique isolates per batch; after excluding genetically identical isolates from the same environmental sampling batch) were obtained from samples collected through the AFP and ES surveillance systems in Pakistan from January 2011 through December 2013 (Table 1). With the exception of five WPV3 isolates detected through AFP surveillance (2011–2012), all detected wild poliovirus was type 1.

**Table 1:** Number of wild poliovirus (WPV) positive isolates by year, serotype, and surveillance system–Jan 2011 –Dec 2013.

	AFP Surveillance^1^		Environmental Surveillance (ES)	Total
*Year*	*WPV1*	*WPV3*	*WPV1*	*WPV1/WPV3*
**Total (2011–2013)**	**361**	**5**	**761**	**1,127**
2011	210	2	370	582
2012	60	3	219	282
2013	91	0	172	262

**Abbreviations:** AFP: acute flaccid paralysis; ES: Environmental surveillance WPV1: Wild poliovirus serotype 1; WPV3: Wild poliovirus serotype 3

^1^ Includes any isolate positive for WPV collected through AFP surveillance (i.e., 36 of 366 isolates are from contacts of AFP cases), and therefore, totals are different case counts reported to WHO for the same time period.

**Fig 1:**
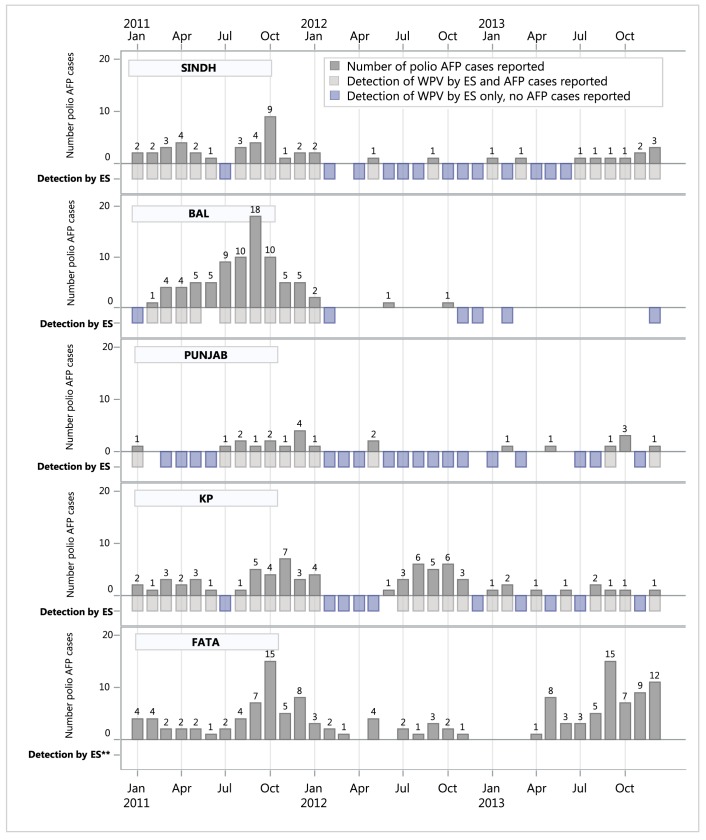
Number of polio AFP cases reported and detection of wild poliovirus (WPV) by environmental surveillance by province in Pakistan by month and year–January 2011 through December 2013*. *Three additional polio AFP cases were reported from Gilgit-Baltistan; One in May 2011 and two in Sept 2012. **From 2011–2013, ES was conducted at sites in Khyber Pakhtunkwa, Punjab, Sindh and Balochistan. There were no ES sites in Gilgit-Baltistan or Federally Administered Tribal Areas. **Abbreviations**: AFP: Acute Flaccid Paralysis; ES: Environmental Surveillance; BAL: Balochistan; FATA: Federally administered tribal areas; GB: Gilgit- Baltistan; KP: Khyber Pakhtunkhwa; PUN: Punjab; SIN: Sindh; WPV: Wild poliovirus.

In the first seven months of 2011, all four provinces conducting ES (Sindh, Balochistan, Punjab, and Khyber Pakhtunkhwa) had at least one month where ES detected poliovirus in sewage samples, but no AFP cases were reported from that province for that month, suggesting silent transmission in these areas (Fig 1). In the latter half of 2011, these provinces experienced an increase in the number of reported polio AFP cases; this suggests that ES detected virus circulation first in Balochistan and before most symptomatic cases of paralysis in Punjab and continued to detect circulation in these areas during 2012–2013, as evidenced by entire provinces without AFP cases, but detecting WPV in sewage isolates (Fig 1).

### Orphan analysis

With AFP surveillance alone, 28 of 366 (7.7%) AFP isolates were orphans (Table 2) ranging from 96.8%-98.4% similar in VP1 capsid nucleotide sequence to any previously detected isolate. Of these 28 isolates, 18 were reported from provinces conducting ES, while the remaining 10 were from provinces without ES sites and two were WPV3. With both AFP surveillance and ES, 10 of these 28 AFP orphans (36%), were linked through ES isolates and no longer considered orphans (28 orphans for AFP surveillance only v. 18 AFP orphans with AFP surveillance plus ES) (Table 2). Of these 10 linked AFP orphans, nine were reported from provinces with ES sites.

**Table 2:** Number of orphan isolates* detected by acute flaccid paralysis surveillance (AFP) only, environmental surveillance (ES) only, and ES and AFP surveillance in conjunction–Jan 2011 –Dec 2013.

Surveillance scenario^1^:	Orphans Detected by AFP-S	Orphans Detected by ES	Total Orphans Detected	Total Isolates	Percent Orphans^3^
1) AFP surveillance only	28	-	28	366	7.7%
2) ES only	-	28	28	761	3.7%
3) Both AFP surveillance & ES^2^	18	19	37	1,127	3.3%

* Orphan viruses are defined as isolates that are ≥1.5% different in the VP1 capsid nucleotide sequence from any isolate previously detected prior to that specimen collection date.

**Abbreviations:** AFP: acute flaccid paralysis; AFP-S: acute flaccid paralysis surveillance; ES: Environmental surveillance

^1^ Each line in the table represents the number of orphan isolates detected considering the listed type of surveillance.

^2^ Includes viruses that remain orphans because there is no AFP isolate or ES isolate within 1.5% of the nucleotide sequence of the VP1 capsid of that virus. 19 (10 AFP and 9 ES) isolates are no longer orphans overall when considering both ES and AFP.

^3^ Orphans detected as a percentage of total isolates for each respective surveillance type

Of the remaining 18 AFP orphan isolates detected by AFP surveillance alone, 10 (55.6%) had more closely related isolates detected by ES than detected by AFP surveillance, but remained orphans when considering both AFP surveillance and ES (i.e., still greater than 1.5% different, but more closely related to an ES isolates than any AFP isolate). Additionally, when considering both AFP and ES surveillance, 19 environmental orphan isolates (range: 95.0% - 98.4% similarity) were detected that were undetected by AFP surveillance (Table 2).

Overall, orphan viruses constituted a lower proportion of all strains when considering ES alone and AFP surveillance plus ES (3.7% and 3.3%, respectively). When considered independently, ES detected a lower proportion of orphan isolates than did AFP surveillance alone (3.7% vs. 7.7%, respectively) (Table 2).

### Circulation analysis

Of the 346 genetically-unique polio cases, 197 (56.9%) had genetically-similar (≥99.0%) preceding circulation detected by both AFP surveillance and ES (Table 3), however, 51 (14.7%) polio cases had no preceding detection by either AFP surveillance or ES. There were 35 (10.1%) polio cases with preceding circulation detected by ES only and 63 (18.2%) cases with preceding circulation detected by AFP only. The relative number of polio cases with preceding circulation detected by both AFP surveillance and ES was lower in 2013 (29.9%) compared to both 2011 (63.7%) and 2012 (74.1%). Conversely, the number and proportion of cases with circulation detected by AFP only was highest in 2013 (Table 3).

**Table 3:** Percentage of Pakistan polio cases* with genetically similar** preceding circulation detected by surveillance system type and year–Jan 2011 –Dec 2013.

		Surveillance system(s) detecting genetically-similar circulation before symptom onset of polio case^1^			
	Total polio cases*	Both AFP-S & ES	ES only^2^	AFP-S only^3^	No detection before case onset^4^
YEAR	*n*	*n (row %)*	*n (row %)*	*n (row %)*	*n (row %)*
**Overall**	**346**	**197 (56.9%)**	**35 (10.1%)**	**63 (18.2%)**	**51 (14.7%)**
2011	201	128 (63.7%)	19 (9.5%)	29 (14.4%)	25 (12.4%)
2012	58	43 (74.1%)	3 (PL5.2%)	2 (3.5%)	10 (17.2%)
2013	87	26 (29.9%)	13 (14.9%)	32 (36.7%)	16 (18.4%)

*28 of 361 total polio cases (11 pairs and 2 triplets) had 100% identity in VP1 capsid nucleotide sequence with one or two other isolates. These pairs and triplets were considered together, resulting in 346 genetically-unique polio case isolate sequences for analysis.

**We defined genetically-similar isolates as isolates with ≥99.0% identity in VP1 capsid nucleotide sequence to the polio case isolate

**Abbreviations:** AFP: acute flaccid paralysis; AFP-S: acute flaccid paralysis surveillance; ES: Environmental surveillance

^1^ Categories are mutually exclusive

^2^ Circulation detected by ES only, no AFP isolates circulating before symptom onset date of polio case

^3^ Circulation detected by AFP surveillance only, no ES isolates circulating before symptom onset date of polio case

^4^ No circulation detected within 99.0% identity. Of these 51 polio cases, 18 are also 'orphans' as defined in Table 2 above (no preceding virus detected within 98.5% similarity in VP1 capsid sequence).

Overall, ES detected circulation first for more polio cases than did AFP surveillance (n = 200, 57.8% vs. n = 95, 27.5%), with more consistent detection for polio cases reported from provinces conducting ES than in those not conducting ES (Table 4). For the 213 polio cases reported from provinces conducting ES, 149 (70.0%) had first detection by ES and 33 (15.5%) had first detection by AFP surveillance. For polio cases reported from provinces not conducting ES, 62 (46.6%) had first detection by AFP and 51 (38.4%) had first detection by ES; for these 51 isolates, ES detection occurred before detection by AFP surveillance, but was detected at ES sites in other provinces. For the 149 cases that had first detection by ES and were reported from provinces conducting ES, 113 (75.8%) had first detection in the same province as the polio case, while the remaining 36 cases had their first detection by ES at sites in other provinces.

**Table 4:** Type of surveillance system first detecting genetically-similar* circulation for each Pakistan polio case** by year, provincial environmental surveillance– 2011–2013.

		Type of surveillance system first detecting circulation^1^		
	Total polio cases*	ES	AFP-S	No detection before case onset^2^
YEAR	*n*	*n (row %)*	*n (row %)*	*n (row %)*
**Overall**	**346**	**200 (57.8%)**	**95 (27.5%)**	**51 (14.7%)**
*ES in Province*^*3*^	*213*	*149 (70*.*0%)*	*33 (15*.*5%)*	*31 (14*.*6%)*
*No ES in Province*^*4*^	*133*	*51 (38*.*4%)*	*62 (46*.*6%)*	*20 (15*.*0%)*
**2011**	**201**	**128 (63.7%)**	**48 (23.9%)**	**25 (12.4%)**
*ES in Province*^*3*^	*146*	*102 (69*.*9%)*	*24 (16*.*4%)*	*20 (13*.*7%)*
*No ES in Province*^*4*^	*55*	*26 (47*.*3%)*	*24 (43*.*6%)*	*5 (9*.*1%)*
**2012**	**58**	**41 (70.7%)**	**7 (12.1%)**	**10 (17.2%)**
*ES in Province*^*3*^	*39*	*29 (74*.*4%)*	*4 (10*.*3%)*	*6 (15*.*4%)*
*No ES in Province*^*4*^	*19*	*12 (63*.*2%)*	*3 (15*.*8%)*	*4 (21*.*1%)*
**2013**	**87**	**31 (35.6%)**	**40 (46.0%)**	**16 (18.4%)**
*ES in Province*^*3*^	*28*	*18 (64*.*3%)*	*5 (17*.*9%)*	*5 (17*.*9%)*
*No ES in Province*^*4*^	*59*	*13 (22*.*0%)*	*35 (59*.*3%)*	*11 (18*.*6%)*

*Genetically-similar isolates were defined as isolates with ≥99.0% identity in VP1 capsid nucleotide sequence to the polio case isolates

**28 of 361 total polio cases (11 pairs and 2 triplets) had 100% identity in VP1 capsid nucleotide sequence with one or two other isolates. These pairs and triplets were considered together, resulting in 346 genetically-unique polio case isolate sequences for analysis.

**Abbreviations:** ES: Environmental Surveillance; AFP: Acute Flaccid Paralysis; AFP-S: Acute Flaccid Paralysis surveillance

^1^ For isolates detected before polio case symptom onset, surveillance system that first detected circulating virus within 99% similarity in VP1 capsid

^2^ Neither AFP surveillance nor environmental surveillance detected circulation preceding symptom onset for polio case

^3^ Polio case was reported from a province conducting environmental surveillance (i.e. Balochistan, Sindh, Punjab, Khyber Pakhtunkhwa)

^4^ Polio case was reported from a province not conducting ES (i.e., Federally Administered Tribal Areas, Gilgit Baltistan)

ES detected circulation first in the majority of polio cases in 2011 and 2012 (128 of 201, 63.7% and 41 of 58, 70.7%, respectively). However in 2013, when most polio cases were reported from FATA, circulation was first detected for more polio cases by AFP surveillance than ES (40 of 87, 46.0% vs. 31 of 87, 35.6%, respectively) (Table 4).

**Fig 2:**
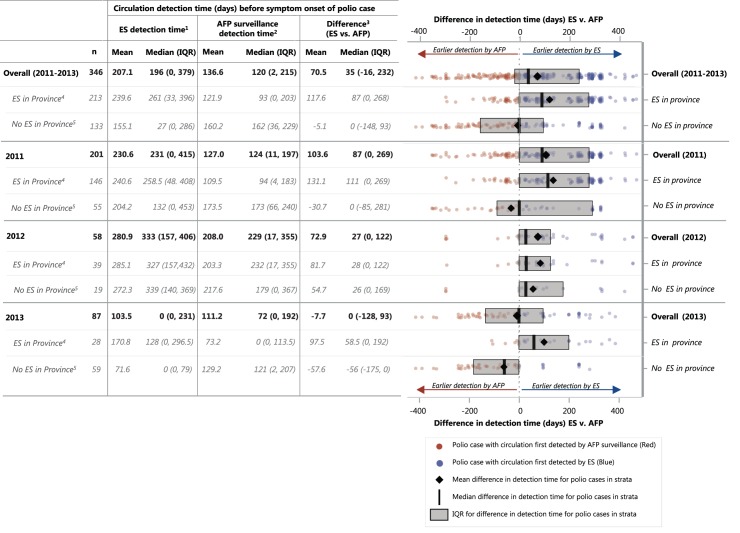
Surveillance system circulation detection time of genetically-similar isolates* before polio case onset and difference in detection time between AFP and environmental surveillance overall, by year and by province environmental surveillance for Pakistan polio cases**– 2011–2013. *Genetically-similar isolates were defined as isolates with ≥99.0% identity in VP1 capsid nucleotide sequence to the polio case isolate. **28 of 361 total polio cases (11 pairs and 2 triplets) had 100% identity in VP1 capsid nucleotide sequence with one or two other isolates. These pairs and triplets were considered together, resulting in 346 genetically-unique polio case isolate sequences for analysis. **Abbreviations**: ES: Environmental Surveillance; AFP: Acute Flaccid Paralysis; AFP-S: Acute Flaccid Paralysis surveillance. ^1^ Time (days) from first isolate within 99% of VP1 capsid detected by environmental surveillance to symptom onset date for polio case. ^2^ Time (days) from first isolate within 99% of VP1 capsid detected by AFP surveillance to symptom onset date for polio case. ^3^ Number of days sooner that environmental surveillance detected circulation before AFP surveillance; Negative numbers indicate AFP detection preceding ES detection and positive numbers indicate ES detection preceding AFP detection. ^4^ Polio case was reported from a province conducing environmental surveillance (i.e. Balochistan, Sindh, Punjab, Khyber Pakhtunkhwa). ^5^ Polio case was reported from a province not conducting ES (i.e. Federally Administered Tribal Areas, Gilgit Baltistan).

Overall, ES detected circulation nearly 71 days sooner on average for each polio case than did AFP surveillance (Fig 2). ES detected genetically-similar circulating virus for an average of 207.1 days before the polio case’s symptom onset date, while AFP surveillance detected circulation for 136.6 days before symptom onset date. For polio cases reported from provinces conducting ES, earlier detection by ES was more pronounced, with ES detecting genetically-similar circulation 117.6 days on average before circulation was detected by AFP surveillance (239.6 days of detection time by ES prior to the case’s symptom onset vs. 121.9 days for AFP surveillance). For polio cases reported from provinces not conducting ES, opposite results were observed, where circulation was detected by AFP surveillance more than five days on average before ES sites in another province detected circulation similar to that polio case. Mean detection time for both AFP and ES was substantially lower in 2013 as compared to 2011–2012. Mean detection by ES and AFP were 230.6 days and 127.0 days, respectively in 2011 compared to 103.5 days and 111.2 days, for ES and AFP surveillance respectively in 2013 (Fig 2).

## Discussion

ES provides a system for routine surveillance of poliovirus circulation independent of clinical disease (AFP). This study examines the contribution of ES as a supplement to AFP surveillance in Pakistan during a period of extensive WPV circulation. Because the two surveillance systems use completely different approaches to finding poliovirus, it is difficult to directly compare performance features. By using virologic results, it is possible to infer qualitative and quantitative benefits from supplementing AFP surveillance with ES. Throughout our study period, standard indicators for quality of AFP surveillance met or exceeded global standards in Pakistan (sensitivity of surveillance >2.0 cases of AFP detected per 100,000, and more than 80% of AFP cases with two adequate stool specimens) [23, 29, 32–37]. Even in the presence of high quality AFP surveillance, through several approaches, the analysis demonstrates the average net benefits of ES to detect circulation earlier than or missed by AFP surveillance.

### Benefits of ES

The first measure of comparison examined was the frequency of ‘orphan’ viruses. Because these viruses are detected after a significant period of replication, they are assumed to represent undetected circulation. The observed reduction in number of orphan viruses detected when considering ES as a supplement to AFP, and the additional detection of ES orphans that would have otherwise been missed, illustrate incremental benefits of supplementary ES over AFP surveillance alone.

For 10% of polio cases, detection of preceding WPV circulation was limited to ES and not detected by AFP surveillance. Even for polio cases with preceding circulation detected by both ES and AFP surveillance, ES detected circulating wild poliovirus sooner. On average, ES detected virus circulation more than two months before detection by AFP surveillance, finding genetically-similar virus circulating on average more than 200 days before the polio case’s symptom onset date. This is consistent with previous, separate analyses of these data in Pakistan where transmission during low-season and reintroductions were detected by ES in the absence of clinical cases [28]. ES was most effective in detecting circulation for polio cases reported from provinces conducting ES, with detection 118 days sooner than AFP surveillance. This time advantage offered by ES in Pakistan was and continues to be used to characterize poliovirus transmission and to appropriately focus resources and response efforts such as supplemental immunization campaigns. For example, after ES isolation of WPV in Lahore, Punjab, public health officials used continued vaccination efforts to prevent spread throughout the province [11]. The early warning provided by ES paired with AFP surveillance allowed the polio program in Pakistan to strengthen immunization practices, resulting in a substantial decline in polio cases and a geographically narrowing polio epidemic observed during our study period. Our data emphasize the programmatic importance of ES, and corroborate that ES provides early warning of WPV circulation before presentation of AFP cases, allowing for more time to implement control measures and immunization activities, preventing further cases.

While AFP surveillance’s target population is the entire country, ES is targeted to the populations covered by the catchment area of the ES sites. With ES in Pakistan, although inherently limited in population coverage by site selection, a lower proportion of all isolates were orphan viruses, suggesting addition of ES leads to more complete surveillance than AFP surveillance alone. Our data document that viruses circulating in provinces without ES sites can be transmitted to and detected in provinces with ES sites even in the absence of clinical cases. For instance, for 51 of the 130 polio cases reported from FATA and GB, the first detection of genetically-similar virus was at ES sites in other provinces demonstrating the ability of ES to detect cross-province circulation before detection by AFP surveillance. For this analysis, we did not differentiate between AFP surveillance and ES detections that represented indigenous circulation and those that represented importations, as we aimed to summarize on a national scale the differences in detection time for genetically-related viruses of each respective surveillance system regardless of location detected. However, for each individual poliovirus detection, public health response should include consideration of phylogenetic, temporal and geographic information, paired with understanding of population mobility into and out of areas without ES sites could be used to provide early warning of circulation and to inform public health action such as mop-up immunization campaigns in these areas. These strategies have been deployed in India, where detection of WPV at ES sites in Mumbai was used to infer importations from Uttar Pradesh and subsequent supplementary immunization activities in both locations [22]. In addition, genetic data from specimens collected through AFP surveillance has been used to describe the cross-border transmission between Pakistan and Afghanistan, and the inclusion of specimens collected through ES may provide more timely information useful in understanding and controlling the spread of poliovirus across borders [38].

### Limitations of the analysis

As Pakistan is one of only three countries with endemic wild virus circulation, it is unlikely that the observed results would be the same in non-endemic areas, and the benefits of ES in these areas should be explored separately. Circulation analyses have the potential for influence by stochastic processes such as sampling frequency for ES and the case-to-infection ratio for AFP. Because we allowed genetically-similar surveillance isolates from one polio case to also be matches for other polio cases, polio cases could not be considered statistically independent, invalidating the basis for further statistical tests. For this analysis, we chose to use individual polio cases as our unit of analysis when we quantified detection by each respective surveillance system, which may have resulted in double counting of nearly identical cases that are often observed in outbreaks. To assess the impact of this, we also conducted this analysis where these clusters of similar cases were considered together as the unit of analysis, and our results and conclusions were robust to these methods.

In our circulation analysis, some of the prior detections by ES and AFP surveillance occurred in different provinces from the province from which the polio case was reported (i.e., cross-province detection); however, in sensitivity analyses, when we restricted genetically-similar matches to only those detected in the same province as the polio case, our observed results remained consistent–ES detected poliovirus circulating prior to the onset of the polio case months sooner on average than did AFP surveillance. Additional analyses are needed to determine if a single positive environmental isolate is predictive of continued circulation in that district or if repeated positive environmental isolates are needed to suggest sustained transmission in an infected area. This information would be useful in informing action to positive environmental sampling, particularly in cases of a single positive environmental isolate.

### Considerations for Expansion of ES

Detection time advantage of ES over AFP surveillance was observed for cases reported from provinces conducting ES (ES detected circulation 118 days before AFP surveillance), but not observed for cases reported from provinces without ES (AFP detected circulation 5 days before ES). This suggests that the planned expansion of ES in Pakistan should continue to target high-risk areas where cases are likely to occur to achieve more efficiency.

Throughout our study period, the epidemiologic situation in Pakistan evolved and more cases were observed in insecure and inaccessible regions of the country without ES sites. The advantages of ES rely on the configuration of sampling sites, sampling procedures, and the local epidemiology of polio. As such, in the last year of our study period (2013) ES demonstrated the least impact as the majority of polio cases occurred in areas without ES. Potential additional ES in Pakistan may be limited in areas of the country that may benefit most, due to inaccessibility of these regions. Absence of requisite converging sewer networks in many areas may limit implementation or efficiency of ES.

ES sensitivity may vary by site and can be influenced by sampling procedures, laboratory practices, and environmental conditions, and differences between sites are difficult to assess. We had limited ability to assess incremental benefit of additional sites as the number of sites was relatively stable throughout our study period. Benefits of scaling ES are difficult to address since variation in epidemiology and vaccination coverage have potential to impact the analysis. Even with AFP surveillance that meets global standards and supplemental ES in Pakistan, there is still evidence of remaining gaps in surveillance. When considering both AFP surveillance and ES, 37 orphan isolates were detected (3.3% of all isolates considered). Circulation analysis suggested that 15% of polio cases had no preceding detection by either AFP or ES–these gaps need further exploration, including exploration of heterogeneity in surveillance quality, to determine why these gaps exist and how surveillance can be improved.

Even in laboratories already processing AFP clinical specimens, ES sample processing increases the workload and cost for laboratories, and should not occur at the expense of AFP surveillance [39, 40]. ES can be considered only a supplement and not a substitute for AFP surveillance. Results from ES, both positive and negative, may be difficult to interpret, but provide important supplemental information to other surveillance systems for response and containment [6, 11].

Although generally recognized to confer a time advantage over AFP surveillance alone, this analysis serves to quantify the substantial time advantage that supplementary ES provides, detecting circulating viruses on average more than 2 months before detection by AFP surveillance, affording an important window for public health intervention. The time advantage, as demonstrated in this analysis for WPV, suggests that the benefits of ES be explored for other important applications as progress is made towards poliovirus eradication and throughout the transition from OPV to IPV. In final stages of eradication, any detection of poliovirus, including WPV and circulating vaccine derived polioviruses (cVDPVs), will require corrective action. For each poliovirus detection, whether through AFP surveillance or ES, the phylogenetic, temporal, and geographic information can be used to determine whether detections represent importations or indigenous circulation and to inform public health response measures.

## Conclusions

Overall, targeted ES through strategic selection of sites has proven useful for detecting WPV circulation in Pakistan. This demonstrated effectiveness for public health response to WPV circulation supports the proposed plan to scale up ES and suggests that ES be explored for other important applications in the eradication endgame strategy, including detecting cVDPVs, monitoring the switch from OPV to IPV, and certification of a polio-free world. Environmental surveillance should be considered in areas at high risk of sustained poliovirus transmission such as localities in endemic countries and those with a history of frequent importations and sustained transmission.

## Supporting information

S1 Fig Map of environmental sampling sites by year of establishment–Pakistan and Afghanistan, 2011–2013. (PDF)

S2 Fig Circulation detected by AFP surveillance and environmental surveillance for Pakistan polio cases and calculated measures of surveillance system detection and relative performance– 2011–2013. Full figure for 346 genetically- unique polio cases in S3 Fig and phylogenetic tree for a subset of ES and AFP isolates can be found in Alam MM et Al, Figure 3, J Infect Dis, 2014).
(PDF)

S3 Fig Circulation detected by AFP surveillance and environmental surveillance for Pakistan polio cases with unique genetic sequences (n = 346). Further descriptions of figure component definitions and calculated measures shown in Tables 4–6 are shown in S2 Fig. Sequence IDs marked with * are also orphan viruses (no preceding virus detected within 1.5% identity). A phylogenetic tree for a subset of ES and AFP isolates can be found in Alam MM et Al, Figure 3, J Infect Dis, 2014.
(PDF)
