# Supplementary material for: The role of supplementary environmental surveillance to complement acute flaccid paralysis surveillance for wild poliovirus in Pakistan – 2011–2013
Source: PLoS One. 2017 Jul 25;12(7):e0180608. doi: 10.1371/journal.pone.0180608 (PMC5526532; doi:10.1371/journal.pone.0180608)
Supplement: S1 Fig — (PDF) [file pone.0180608.s001.pdf]

S1 Fig. Map of environmental sampling sites by year of establishment – Pakistan and Afghanistan, 2011-2013.

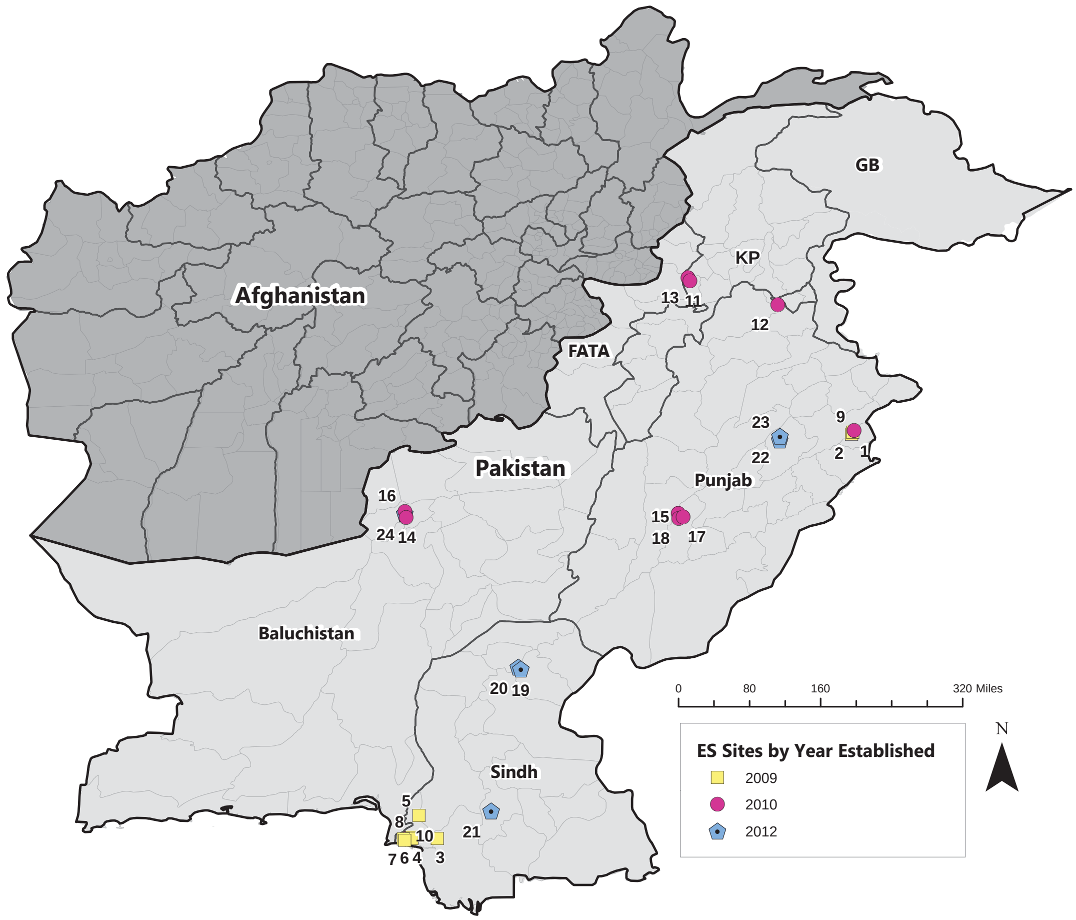

#	ENVIRONMENTAL SURVEILLANCE SITE NAME	PROVINCE (PAKISTAN)	DISTRICT	START YEAR	END YEAR
1	GULSHAN RAVI STATION	PUNJAB	LAHORE	2009	-
2	MULTAN ROAD STATION	PUNJAB	LAHORE	2009	-
3	CHAKORA NULLA	SINDH	KHI.GULSHAN-E-IQBAL TOWN	2009	-
4	RASHID MINHAS RD LAY	SINDH	KHI.GULSHAN-E-IQBAL TOWN	2009	-
5	COMPOSITE SAMPLE	SINDH	KHI.GADAP TOWN	2009	-
6	SAJJAN GOTH	SINDH	KHI.BALDIA TOWN	2009	-
7	KUMHAR WARRA HUB RIV	SINDH	KHI.BALDIA TOWN	2009	2010
8	SOHRAB GOTH	SINDH	KHI.GADAP TOWN	2009	-
9	OUTFALL STATION	PUNJAB	LAHORE	2010	-
10	BALDIA COMPOSITE	SINDH	KHI.GADAP TOWN	2010	-
11	SHAHEEN TOWN	KHYBER PAKHTUNKHWA	PESHAWAR	2010	-
12	SAFDAR ABAD	PUNJAB	RAWALPINDI	2010	-
13	LARA MA	KHYBER PAKHTUNKHWA	PESHAWAR	2010	-
14	JATAK KILLI & TAKHTHANI BY PASS	BALOCHISTAN	QUETTA	2010	-
15	SURAJ MIANI	PUNJAB	MULTAN	2010	-
16	JAM-E-SALFIA	BALOCHISTAN	QUETTA	2010	-
17	ALI TOWN	PUNJAB	MULTAN	2010	-
18	KOTLA ABDUL FATAH	PUNJAB	MULTAN	2010	-
19	MIANI PUMPING STATION	SINDH	SUKKUR	2012	-
20	MAKKA PUMPING STATION	SINDH	SUKKUR	2012	-
21	TULSIDAS PUMPING STATION	SINDH	HYDERABAD	2012	-
22	FAISALABAD COMPOSITE SITE	PUNJAB	FAISALABAD	2012	-
23	ISMAIL PUMPING STATION	PUNJAB	FAISALABAD	2012	-
24	SUR PUL	BALOCHISTAN	QUETTA	2012	-

Abbreviations: KP: Khyber Pakhtunkhwa; GB: Gilgit-Baltistan;FATA: Federally Administered Trial Areas ES: Environmental Surveillance
