# Supplementary material for: The role of supplementary environmental surveillance to complement acute flaccid paralysis surveillance for wild poliovirus in Pakistan – 2011–2013
Source: PLoS One. 2017 Jul 25;12(7):e0180608. doi: 10.1371/journal.pone.0180608 (PMC5526532; doi:10.1371/journal.pone.0180608)
Supplement: S2 Fig — Full figure for 346 genetically- unique polio cases in S3 Fig and phylogenetic tree for a subset of ES and AFP isolates can be found in Alam MM et Al, Figure 3, J Infect Dis, 2014). (PDF) [file pone.0180608.s002.pdf]

S2 Fig. Circulation detected by AFP surveillance and Environmental Surveillance for Pakistan polio cases and calculated measures of surveillance system detection and relative performance – 2011-2013. Full figure for 346 genetically-unique polio cases in S3 Fig and phylogenetic tree for a subset of ES and AFP isolates can be found in Figure 3, Alam MM et Al, J Infect Dis, 2014).

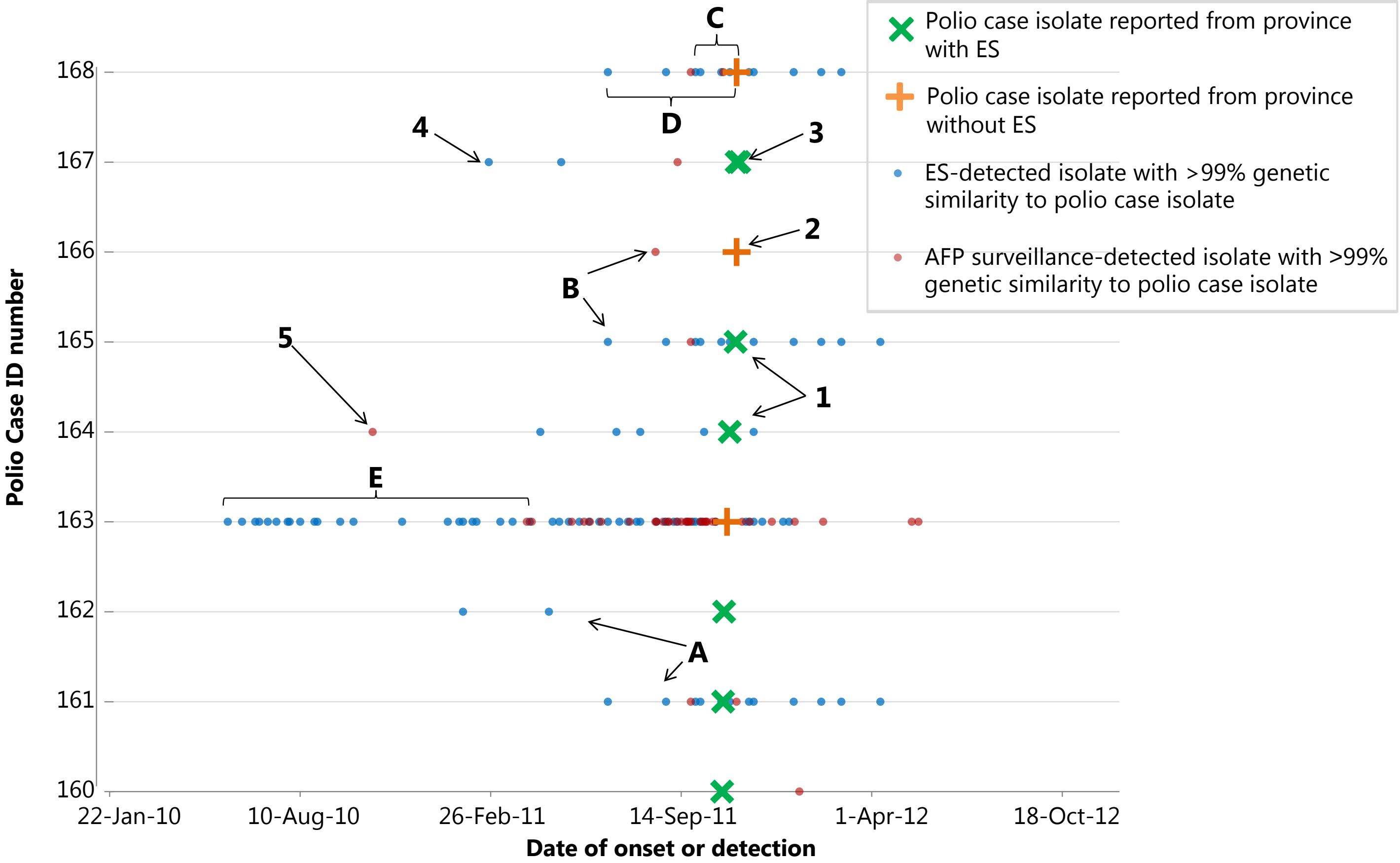

Figure Component	Description
**X-axis**	Date of onset (AFP isolates) or collection date (ES isolates)
**Y-axis**	Unique ID number for polio case isolate, numbered in ascending order by symptom onset date
**1**	(Large green X) Symptom onset date date for polio case reported from a province conducting ES (i.e. Balochistan, Sindh, Punjab, or Khyber Pakhtunkhwa).
**2**	(Large orange +) Symptom onset date date for polio case reported from a province not conducting ES (i.e. Federally Administered Tribal Areas or Gilgit Baltistan).
**3**	(Multiple green X's on one line) Two polio case isolates with identical sequences. Lines with single isolates indicate that that isolate was genetically unique from all other polio case isolates. For identical isolates, symptom onset for the first case is used in calculated measures described below.
**4**	(Blue circles) Any isolate detected by environmental surveillance within >99.0% VP1 similarity to polio case isolates (green X's or orange +'s)
**5**	(Red circles) Any isolate detected by AFP surveillance between >99.0% VP1 sequence identity to polio case isolates (green X's or orange +'s).
**Calculated Measures**	**Description**
**A**	Type surveillance system detecting of preceding circulation (Table 3) –categories are AFP only, ES only, AFP and ES and neither. For polio case 161, both AFP and ES detected circulation, therefore the type of prior circulation is "AFP & ES"; For polio case 162, only ES detected circulation (no AFP isolates detected) and type of prior circulation is "ES only"
**B**	Type of surveillance system first detecting circulation (Table 4) – possible categories are AFP, ES or neither. For example, polio case 166 has AFP surveillance detection first, polio case number165 has ES detection first, and polio case number160 has neither AFP nor ES detection before.
**C**	Circulation time in days detected by AFP surveillance before polio case presents (Table 5). Calculated as the time from first detected AFP isolate (red dot) within ≥99.0% identity to the symptom onset date for the polio case (green X or orange +).
**D**	Circulation time in days detected by environmental surveillance before polio case presents (Table 5). Calculated as the time from first detected environmental isolate (blue dot) within ≥ 99.0% similarity in VP1 capsid to the symptom onset date for polio case isolate (green X or orange +).
**E**	Difference in detection time between AFP surveillance and ES (Table 5). Calculated as time in days from first detected ES surveillance isolate (blue circle) to first detected AFP surveillance isolate (red circle).
**Other Notes**	• Polio cases depicted as lone green X's or orange +'s have no matches within 99% similarity in their VP1 capsid<br>• Complete figure (346 unique polio cases) available in supplementary appendix, figure 3.
