# Supplementary material for: The role of supplementary environmental surveillance to complement acute flaccid paralysis surveillance for wild poliovirus in Pakistan – 2011–2013
Source: PLoS One. 2017 Jul 25;12(7):e0180608. doi: 10.1371/journal.pone.0180608 (PMC5526532; doi:10.1371/journal.pone.0180608)

S3 Fig (Part 1 of 5). Circulation detected by AFP surveillance and Environmental Surveillance for Pakistan polio cases with unique genetic sequences (n=346). Further descriptions of figure component definitions and calculated measures shown in Tables 4-6 are shown in S2 Fig. Sequence IDs marked with * are also orphan viruses (no preceding virus detected within 1.5% identity). A phylogenetic tree for a subset of ES and AFP isolates can be found in Figure 3, Alam MM et Al, J Infect Dis, 2014.

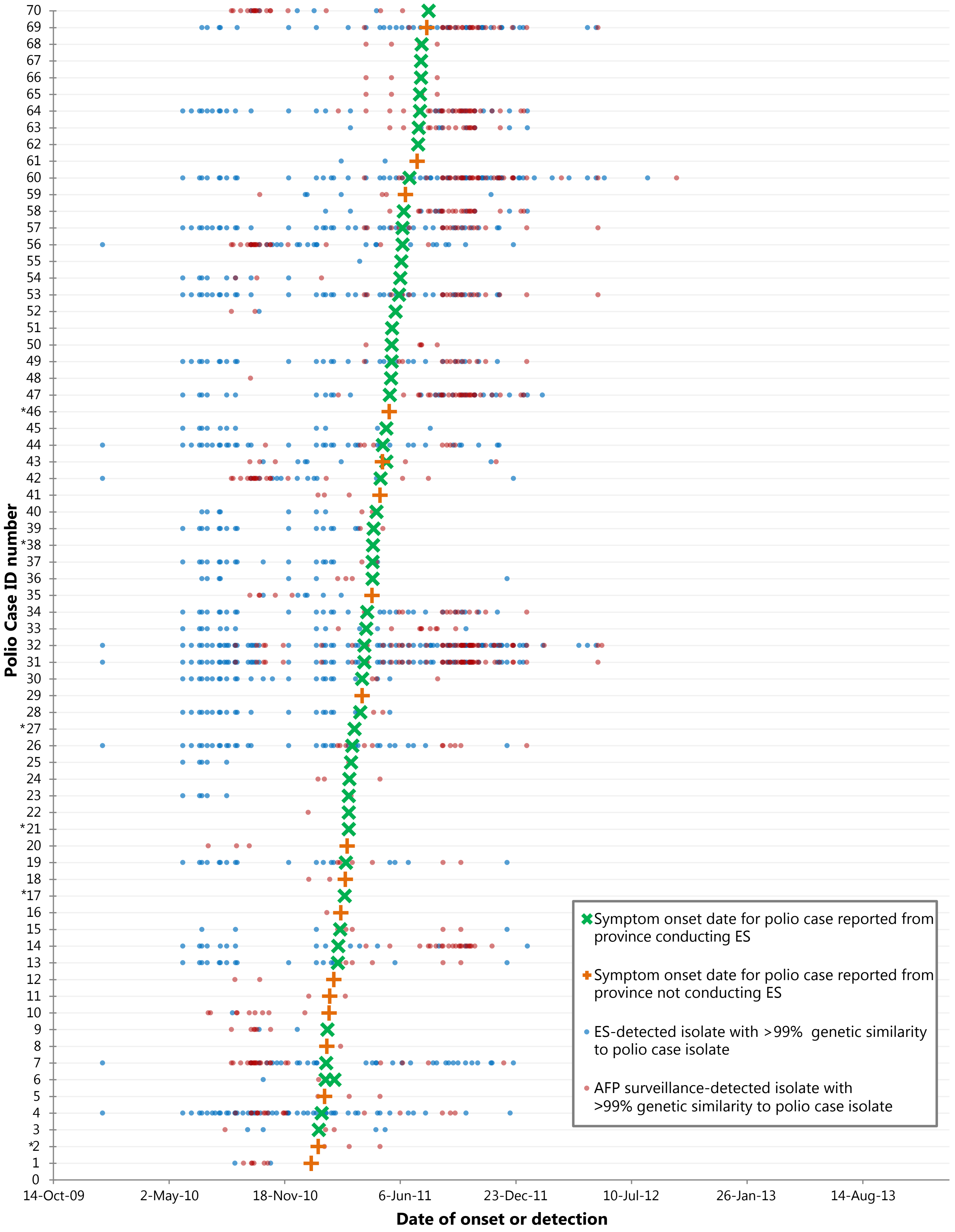

S3 Fig (Part 2 of 5). Circulation detected by AFP surveillance and Environmental Surveillance for Pakistan polio cases with unique genetic sequences (n=346). Further descriptions of figure component definitions and calculated measures shown in Tables 4-6 are shown in S2 Fig. Sequence IDs marked with * are also orphan viruses (no preceding virus detected within 1.5% identity). A phylogenetic tree for a subset of ES and AFP isolates can be found in Figure 3, Alam MM et Al, J Infect Dis, 2014.

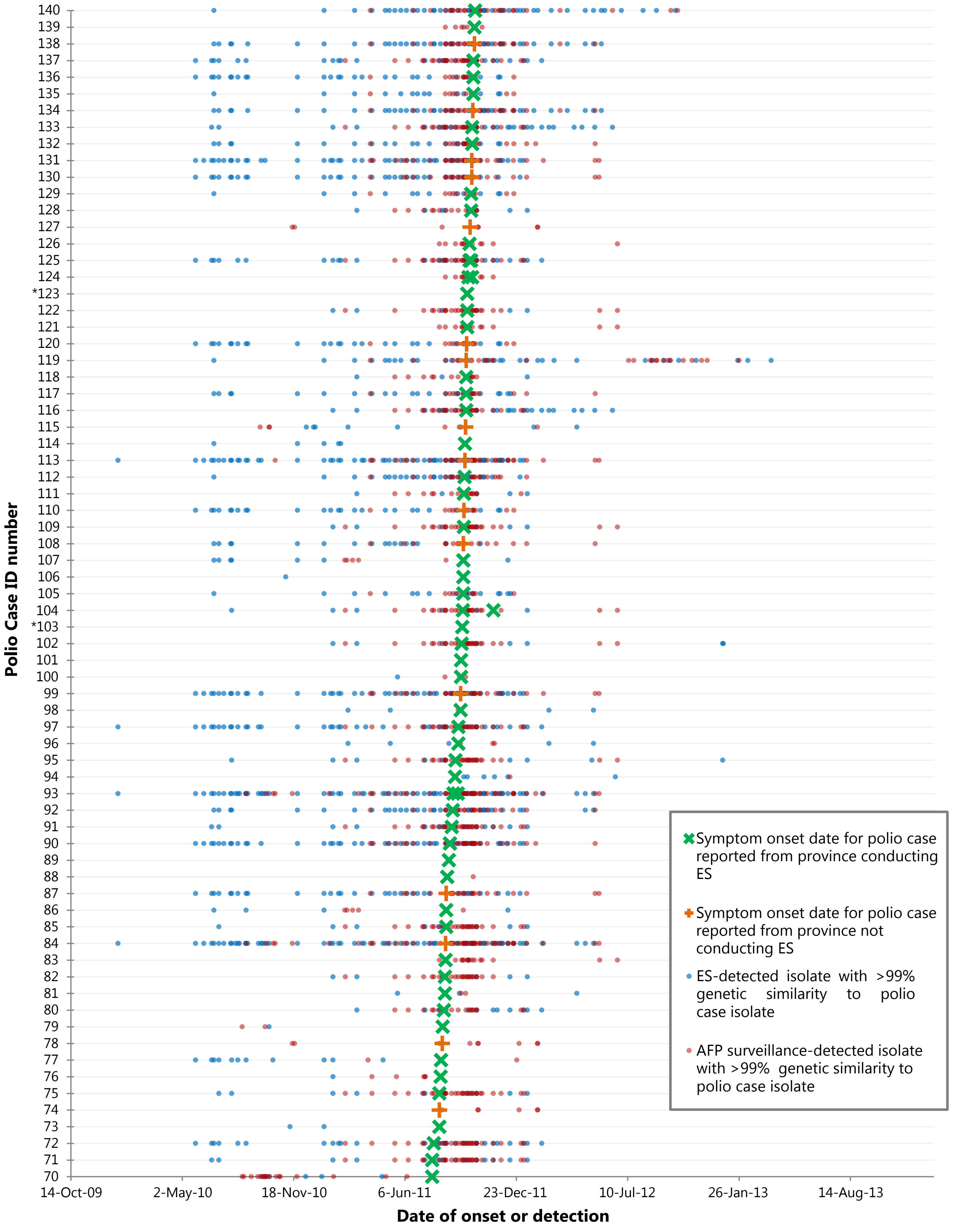

S3 Fig (Part 3 of 5). Circulation detected by AFP surveillance and Environmental Surveillance for Pakistan polio cases with unique genetic sequences (n=346). Further descriptions of figure component definitions and calculated measures shown in Tables 4-6 are shown in S2 Fig. Sequence IDs marked with * are also orphan viruses (no preceding virus detected within 1.5% identity). A phylogenetic tree for a subset of ES and AFP isolates can be found in Figure 3, Alam MM et Al, J Infect Dis, 2014.

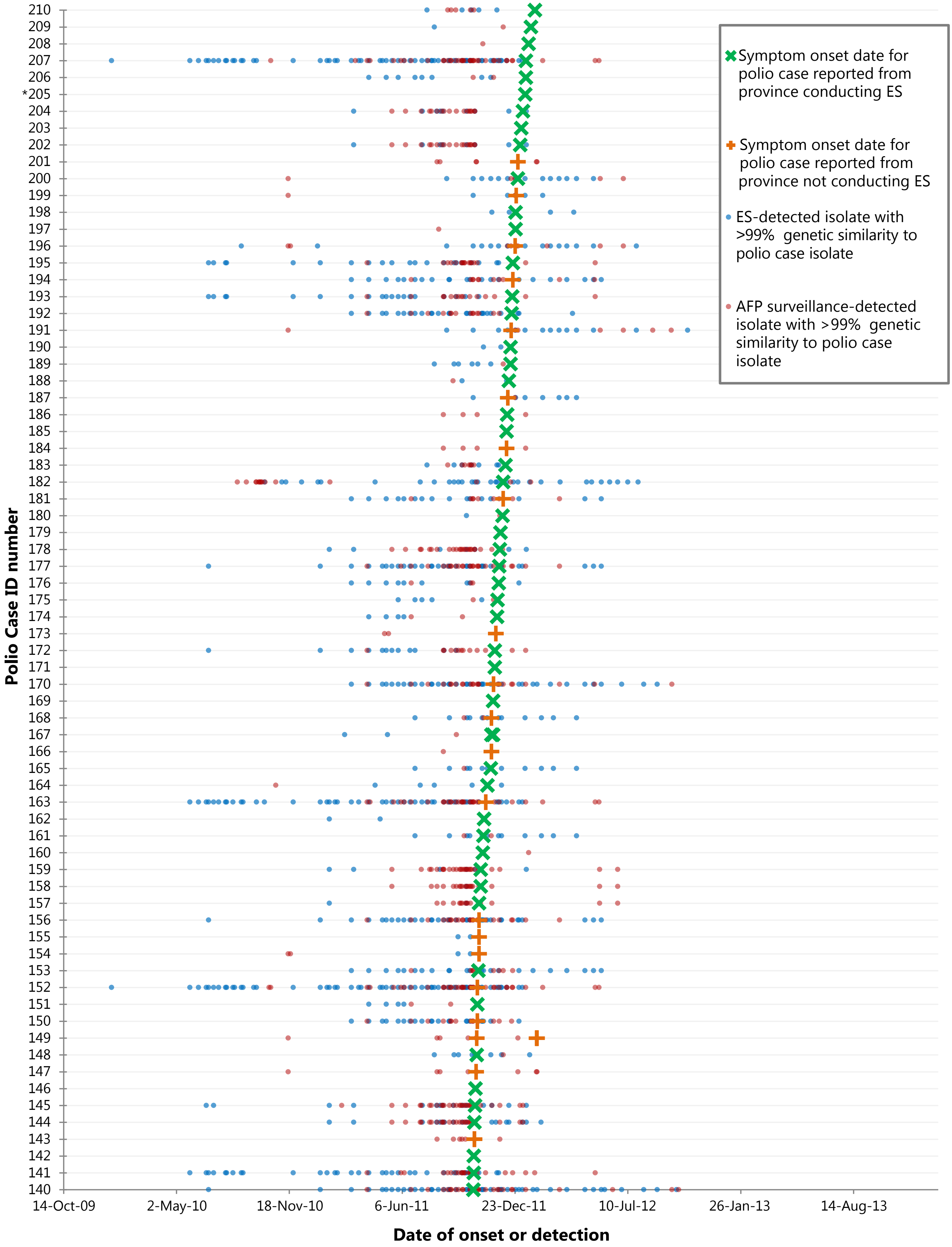

S4 Fig (Part 4 of 5). Circulation detected by AFP surveillance and Environmental Surveillance for Pakistan polio cases with unique genetic sequences (n=346). Further descriptions of figure component definitions and calculated measures shown in Tables 4-6 are shown in S2 Fig. Sequence IDs marked with * are also orphan viruses (no preceding virus detected within 1.5% identity). A phylogenetic tree for a subset of ES and AFP isolates can be found in Figure 3, Alam MM et Al, J Infect Dis, 2014.

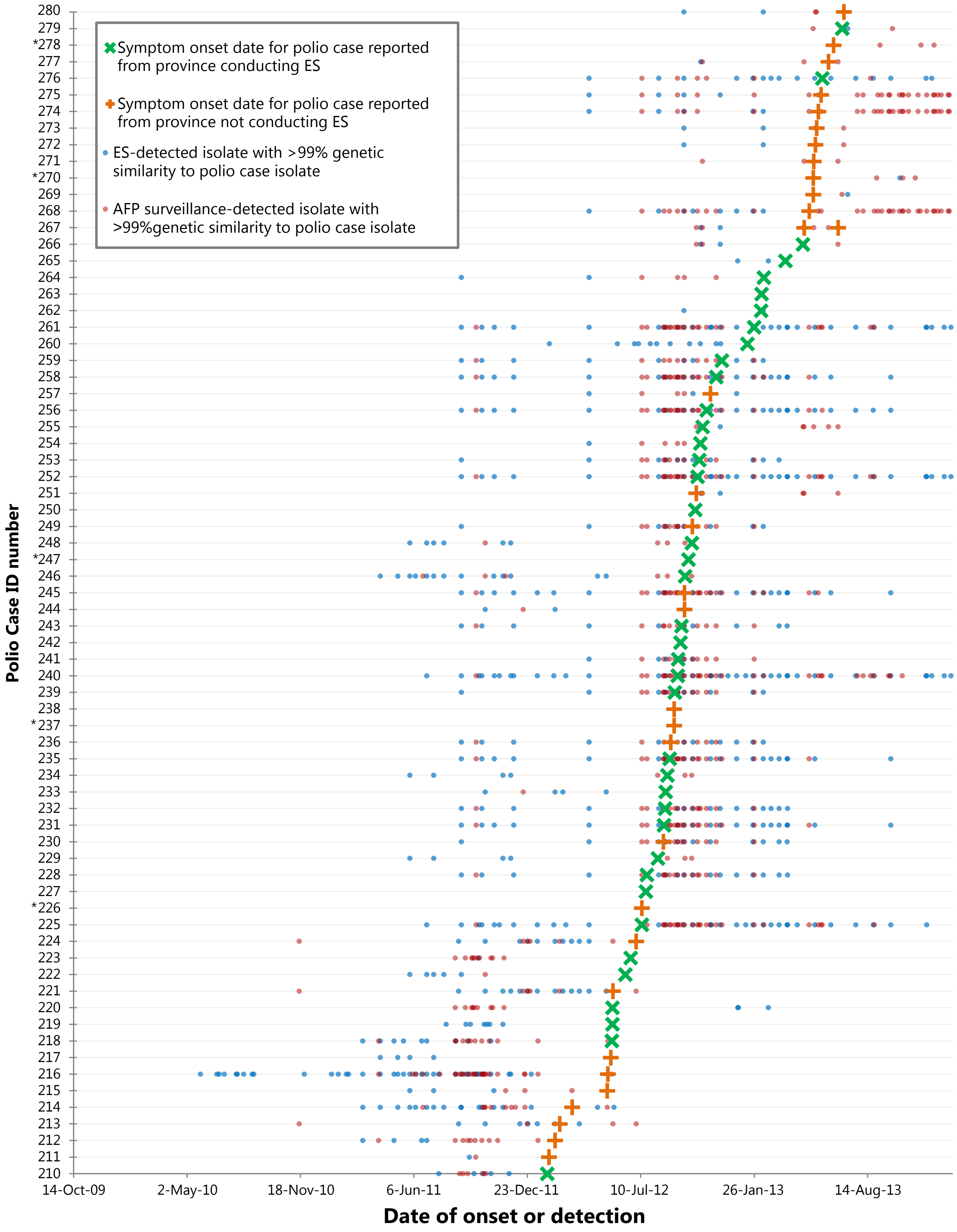

S4 Fig (Part 5 of 5). Circulation detected by AFP surveillance and Environmental Surveillance for Pakistan polio cases with unique genetic sequences (n=346). Further descriptions of figure component definitions and calculated measures shown in Tables 4-6 are shown in S2 Fig. Sequence IDs marked with * are also orphan viruses (no preceding virus detected within 1.5% identity). A phylogenetic tree for a subset of ES and AFP isolates can be found in Figure 3, Alam MM et Al, J Infect Dis, 2014.

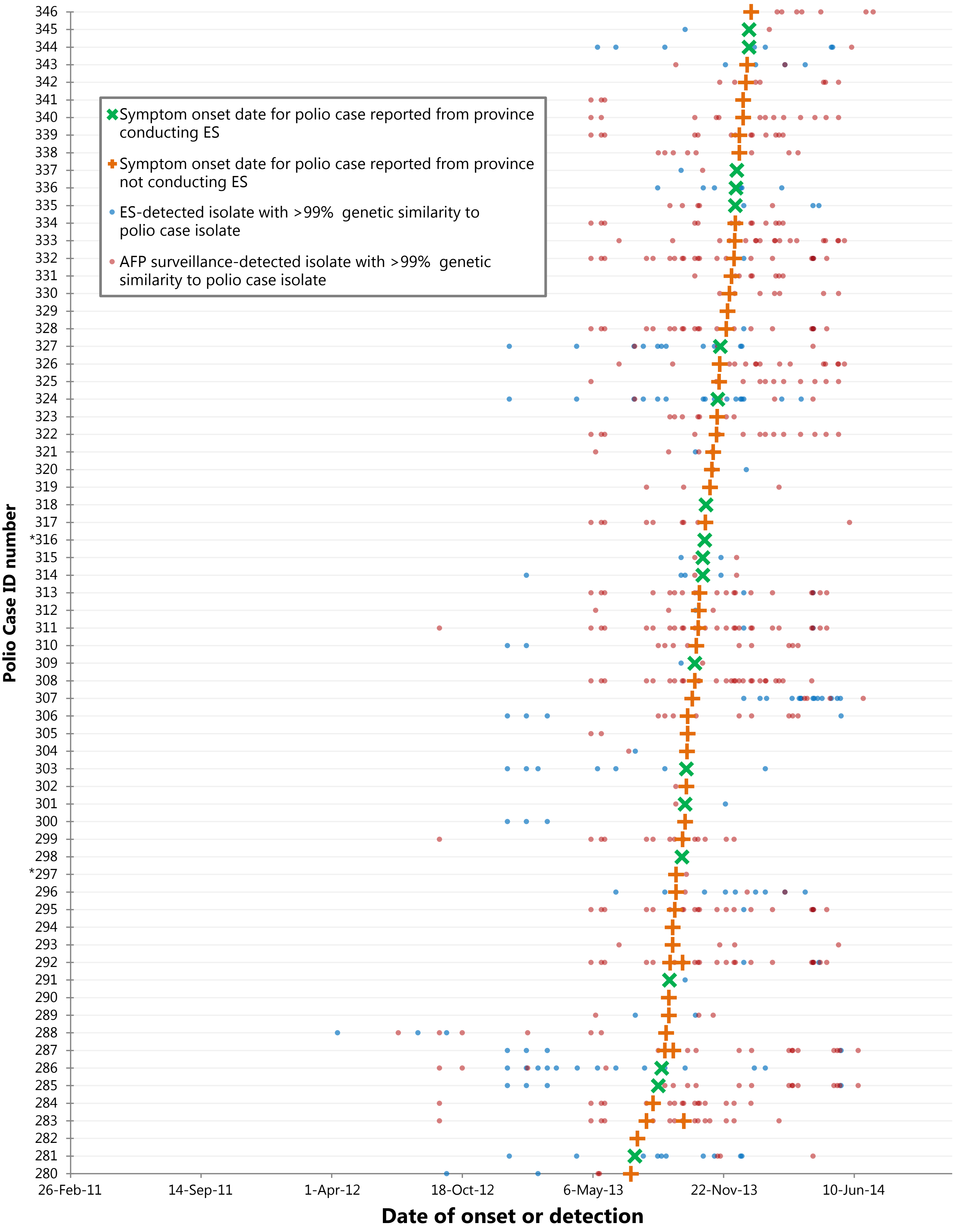

Supplement: S3 Fig — Further descriptions of figure component definitions and calculated measures shown in Tables 4–6 are shown in S2 Fig. Sequence IDs marked with * are also orphan viruses (no preceding virus detected within 1.5% identity). A phylogenetic tree for a subset of ES and AFP isolates can be found in Alam MM et Al, Figure 3, J Infect Dis, 2014. (PDF) [file pone.0180608.s003.pdf]
